# Codon Preference Optimization Increases Heterologous PEDF Expression

**DOI:** 10.1371/journal.pone.0015056

**Published:** 2010-11-30

**Authors:** Anzor G. Gvritishvili, Kar Wah Leung, Joyce Tombran-Tink

**Affiliations:** 1 Department of Neural and Behavioral Sciences, Penn State University College of Medicine, Hershey, Pennsylvania, United States of America; 2 Department of Ophthalmology, Penn State University College of Medicine, Hershey, Pennsylvania, United States of America; University of Edinburgh, United Kingdom

## Abstract

Pigment epithelium-derived factor (PEDF) is widely known for its neurotrophic and antiangiogenic functions. Efficacy studies of PEDF in animal models are limited because of poor heterologous protein yields. Here, we redesigned the human PEDF gene to preferentially match codon frequencies of *E coli* without altering the amino acid sequence. Following de novo synthesis, codon optimized PEDF (coPEDF) and the wtPEDF genes were cloned into pET32a containing a 5′ thioredoxin sequence (Trx) and the recombinant Trx-coPEDF or Trx-wtPEDF fusion constructs expressed in native and two tRNA augmented *E coli* hosts - BL21-CodonPlus(DE3)-RIL and BL21-CodonPlus(DE3)-RP, carrying extra copies of tRNA*^arg,ile,leu^* and tRNA*^arg,pro^* genes , respectively. Trx-PEDF fusion proteins were isolated using Ni-NTA metal affinity chromatography and PEDF purified after cleavage with factor Xα. Protein purity and identity were confirmed by western blot, MALDI-TOF, and UV/CD spectral analyses. Expression of the synthetic gene was ∼3.4 fold greater (212.7 mg/g; 62.1 mg/g wet cells) and purified yields ∼4 fold greater (41.1 mg/g; 11.3 mg/g wet cell) than wtPEDF in the native host. A small increase in expression of both genes was observed in hosts supplemented with rare tRNA genes compared to the native host but expression of coPEDF was ∼3 fold greater than wtPEDF in both native and codon-bias-adjusted *E coli* strains. ΔGs at −3 to +50 of the Trx site of both fusion genes were −3.9 kcal/mol. Functionally, coPEDF was equally as effective as wtPEDF in reducing oxidative stress, promoting neurite outgrowth, and blocking endothelial tube formation. These findings suggest that while rare tRNA augmentation and mRNA folding energies can significantly contribute to increased protein expression, preferred codon usage, in this case, is advantageous to translational efficiency of biologically active PEDF in E coli. This strategy will undoubtedly fast forward studies to validate therapeutic utility of PEDF in vivo.

## Introduction

Pigment epithelium-derived factor (PEDF) is a polypeptide functionally characterized in the nervous system as a protein with neuroprotective and antiangiogenic activities [1], [2]. Its major synthesis and secretion in the eye is by the retinal pigment epithelium (RPE) [1]. In humans, PEDF is found in quantities of 144 nM in the interphotoreceptor matrix (IPM), 21 nM in the vitreous humor and 6 nM in the aqueous humor of the eye [1] and in approximately 8 to 24 µg/ml (mean (SD) 14.3 µg/ml) in serum [3]. The PEDF gene encodes 418 amino acid residues and its calculated molecular weight is 46 kDa [4]. PEDF contains a single carbohydrate side chain that increases its apparent molecular weight to 50 kDa on polyacrylamide gels.

The importance of this protein to biological systems has been demonstrated in a many in vitro and in vivo models. Some of these include studies which showed the neurotrophic and cell survival actions of the protein on a wide range of neurons in the CNS as well as the photoreceptor and retinal ganglion cells of the eye [1]. The PEDF gene is highly conserved in evolution and resides on human chromosome 17p13 [5]–[7]. The neurotrophic activity of PEDF is localized to the N terminal part of the protein, corresponding to amino acid residues 78–121 [8]. The multifunctional actions of the molecules is separated on distinct peptide domains which appear to activate different receptors including the laminin receptor [9], VEGF R1 [10], and the ATLG receptor [11]. Its effectiveness as an angiogenesis inhibitor has been defined by its ability to inhibit retinal endothelial cell (EC) growth and migration and the abnormal growth of blood vessels in eyes subjected to ischemic injury, hyperoxia, exitotoxicity and axotomy [1], [12], [13]. The levels of PEDF decreases in many angiogenic and neurodegenerative eye diseases, suggesting it is important to the health and function of the eye [2], [14], [15]. Not only is it important to ocular diseases, several studies have reported that tumor burden of some cancers can be reduced by the antiangiogenic and differentiation properties of PEDF [16]–[25]. The 50 kDa protein has structural and sequence homology with members of the serpin gene family of proteinase inhibitors and contains a reactive center loop (RCL) that is characteristic of this family [4], [26]. The crystal structure of PEDF shows a unique asymmetric charge amino acid distribution, an unusual concentration of positively charged amino acid residues which might be of functional importance, and several possible functional domains including two binding sites with extracellular matrix molecules [1], [26], [27].

Although there is some information about the structure of this protein and its functional activities, many studies are limited lack of readily available protein due to the poor yields in *Escherichia coli* (*E.coli*). Large amounts of PEDF are required for functional studies in vivo that pertains to tumor growth and metastasis, vascular diseases, degenerations in the CNS and for structural analysis of protein-ligand interactions. PEDF has also been identified as a niche signal for neural stem cell self-renewal and can induce cone photoreceptor markers in primary retinal neural aggregates [28]–[30]. Here we used codon optimization strategies to improve the expression of PEDF in *E coli*. Codon optimization, a genetic technique used to achieve optimum expression of a foreign gene in a host's cell system is achieved by replacing existing codons of a specie with a set of more suitable host codons [31]–[34]. Bacteria and mammals prefer to use different codons [34] so many mammalian genes such as PEDF are poorly expressed in bacteria. Alternatively, heterologous expression can be improved by supplying the host with extra copies of rare tRNA genes [35]. The results from our work suggest that codon optimization of PEDF for bacterial expression increases the yield of the protein above the expression yields of the non-optimized gene in both native *E. coli* strains and those supplemented with rare tRNA genes. coPEDF is as functionally active as wtPEDF in promoting neurite outgrowth from SHSY5Y neuroblastoma cells, in protecting these cells from hydrogen peroxide toxicity, and in preventing endothelial tube formation.

## Materials and Methods

All tissue culture reagents were obtained from Invitrogen –Gibco (Gaithersburg, MD) and the human SHSY5Y neuroblastoma cells from ATCC (Mannassas, VA). The restriction enzymes XhoI, KpnI, BstbI, HindIII and factor Xα protease were purchased from New England BioLabs (Beverly, MA). The BL21Star(DE3) and TOP10 chemically competent cells were obtained from Invitrogen life technologies (Carlsbad, CA) and BL21-CodonPlus(DE3)-RIL and BL21-CodonPlus(DE3)-RP competent cells from Stratagene (La Jolla, CA). The pET32a vector and Ni-NTA His-Bind resin was purchased from Novagen (Madison, WI) and Goat anti rabbit conjugated IgG from Jackson ImmunoResearch Laboratories (West Grove, PA). All PCR primers were synthesized by the HMC Molecular Genetics Core Facility at PSU.

### Codon optimization of human PEDF cDNA for Expression in *E coli*

The human full-length PEDF DNA (1257 bp) was codon-optimized for bacterial expression using Genscript OptimumGene™ design platform, which employs a unique algorithm and proprietary codon usage table. Modifications were made throughout the sequence in approximately 25.5% of the DNA. Codon optimized PEDF (coPEDF) was successfully assembled using overlapping PCR (Genscript). The pCMV6-XL5 plasmid (Origene, MD) containing the entire wild type PEDF (wtPEDF) DNA was used to obtain the human wild type PEDF (wtPEDF) DNA coding sequence of 1257 bp. The free energies of the genes (ΔG) were computed using the online RNA folding program “mfold” (http://www.bioinfo.rpi.edu/applications/mfold [36].

### Construction of Plasmids for Expression of coPEDF and wtPEDF

The synthetic version of the gene (coPEDF) and wtPEDF DNA containing the entire PEDF ORF with its synthetic and naturally occurring amino terminus (Met^1^-Pro^418^) were cloned into the pET32a vector (Novagen) using the KpnI and Hind III restriction sites. The vector appends an N-terminal polyhistidine tag to the protein for Ni-NTA metal-affinity chromatography purification, an upstream protein folding enhancement thioredoxin (Trx) fragment of 109 amino acids, and a cleavable Trx.Tag coding sequence. The synthetic and wtPEDF plasmid constructs, pET32a-coPEDF and pET32a-wtPEDF were used to transform TOP10 chemically competent cells (invitrogen). Several transformed colonies were isolated from ampicillin agar plates and grown in 3 ml LB medium containing 100 ug/ml ampicillin. coPEDF and wtPEDF plasmid DNAs were isolated from minipreps and enzymatically digested with KpnI, XhoI and BstBI, HindIII (Biolabs) respectively. The digested DNA were then separated using 1% agarose gels and plasmids containing the DNA product size (1257 bp) were subjected to sequencing (HMC Molecular Genetics Core Facility at PSU).

### Expression of coPEDF and wtPEDF

5 nanograms of pET32a-coPEDF and pET32a-wtPEDF plasmid constructs were used to transform 50 ul *E. coli* BL21Star(DE3) (invitrogen) for 30 min at 4°C, followed by 30 s at 42°C. To correct heterologous expression problems that could be caused by codon bias, the wtPEDF plasmid was also expressed in BL21-CodonPlus(DE3) cells overexpressing rare tRNAs genes for codons most frequently used in GC-rich mammalian genomes. The coPEDF construct was also expressed in these cells to determine whether codon usage alone increased heterologous gene expression.

Plasmids were expressed in two BL21-CodonPlus(DE3) cells: BL21CodonPlus(DE3)-RIL cells supplemented with extra copies of the *argU, ileY, and leuW* tRNA genes (BL21-CodonPlus(DE3)-RIL*^argU, ileY, and leuW^*), which recognize AGA/AGG, AUA, and CUA codons, respectively and in the BL21-CodonPlus(DE3)-RP competent cells containing extra copies of the tRNA genes, *argU* (BL21-CodonPlus(DE3)-RP*^argU, proL^*) which recognizes the AGA and AGG arginine codons, and *proL* which recognizes the proline codon CCC (Stratagene).

The mixtures were incubated on ice for 2 min then, 250 µl (SOC) medium added for an additional incubation at 37°C for 1 hr with constant shaking at 250 rpm. The cells were plated on ampillicin agar plates and grown at 37°C overnight. Transformed colonies of host cells containing the expression vectors were grown in 1 liter LB medium containing 100 µg/ml ampicilin at 37°C overnight to logarithmic phase. Initial expression of the proteins was induced by 1 mmol/L isopropyl-β-D-triogalactoside (IPTG) for 3 hr at 37° when the OD_600_ nm  = 0.8, 200 µl cells were harvested by centrifugation and pellets resuspended in 25 µl Laemmli Sample Buffer (Bio-Rad). Expressed proteins in the subcellular fractions were detected by SDS-PAGE and western blot analysis. Overexpressing clones from the synthetic and native PEDF gene transformants were selected for optimizing induction conditions, which included time of inducement, concentration of IPTG, and inducement temperature.

For large-scale production, expression was carried out using induction optimized conditions of 0.5 mmol/L IPTG for 5 hrs at 25°C. 1 L cultures of *E-coli* containing the constructs were grown at 37°C in LB medium, 100 µg/ml ampicilin and expression induced when the optical density of the cultures was approximately 0.8 units at OD600 nm. Cells were then harvested by centrifugation at 4000 g for 20 min at 4°C. Bacterial yield was approximately 2.28 g packed *E. Coli cells*/per L of culture. Cell pellets were then lysed in buffer containing 10 mM sodium phosphate, 150 mM sodium chloride, and 2% SDS pH 7.8 and insoluble fractions removed by centrifugation at 12000 g for 30 min. The supernatant was collected and diluted in 50 mM NaPhosphate, 300 mM NaCl, 1 mM DTT, pH 8.0 buffer and dialyzed against same buffer overnight at 4°C.

### Protein purification

Soluble Trx-coPEDF and Trx-wtPEDF were isolated by Ni-NTA metal-affinity chromatography. The dialysate was loaded onto a Ni-NTA His-binding resin (Novagen) and column equilibrated with 1× Ni-NTA Binding Buffer containing 50 mM NaH_2_PO_4_, 300 mM NaCl, 10 mM imidazole, pH 8.0. The flow through was collected and the column washed with 1× Ni-NTA buffer containing 50 mM NaH_2_PO_4_, 300 mM NaCl, 20 mM imidazole, pH 8.0. Bound His-tagged Trx-coPEDF and Trx-wtPEDF were then eluted with 1× Ni-NTA buffer containing 50 mM NaH_2_PO_4_, 300 mM NaCl, 250 mM imidazole, pH 8.0. Protein fractions were pooled and concentrated using Amicon Ultra Centrifugal Filter Device, with cellulose MWCO of 30 kDa (Millipore, Billenica, MA). The protein fractions were then applied onto Sephadex G-100 gel-filtration column (2.0×50 cm) and equilibrated with buffer containing 10 mM NaH_2_PO_4_, 150 mM NaCl, pH 7.2, 3 mM DTT. Protein fractions were collected and concentrated using an Eppendorf Speed Vac and protein concentration determined using the Bradford assay (Bio-Rad) and PEDF ELISA (Bioproducts, MD). Purity of the proteins was estimated by 10% SDS-PAGE and the molecular weight confirmed by western blot analysis.

coPEDF and wtPEDF were cleaved from the 109 amino acid N-terminal thioredoxin peptide at the Trx.Tag site IIeGluGlyArg using factor Xα (BioLabs). The reaction proceeded ∼6 hrs in buffer containing 100 mM NaCl, 20 mM Tris-HCl, 2 mM CaCl_2_, pH 8.0 at room temperature and factor Xα:protein with ratio (unit/µg) of 1∶50. The protein mixture was dialyzed against 50 mM NaPhosphate, 300 mM NaCl, pH 8.0 buffer and applied twice sequentially onto Ni His-binding resin. The unbound ∼50 kDa cleaved coPEDF and wtPEDF fractions were collected, combined, concentrated, and dialyzed against PBS buffer containing 10 mM NaPhosphate, 150 mM NaCl pH 7.8 using Spectrapor dialysis membranes MWCO: 3 500 Da. The purified protein was stored at 20°C. Protein purity and molecular weight were confirmed by SDS-PAGE and western blot analyses. An aliquot of the proteins were taken for mass spectrometry for comparison and for UV and CD spectral analyses.

### ELISA assay

coPEDF and wtPEDF concentrations in bacterial lysates and after purification were estimated by ELISA assay (BioProducts MD, Middletown, MD). Briefly, protein samples at 1∶10 or 1∶100 dilutions were incubated in human PEDF antibody coated 96well plates for 1 h at 37°C. After several washes, a PEDF detector antibody was added and incubated for an additional hour at 37°C followed by washing and incubation with streptavidin-conjugated peroxidase for 30 min at 37°C. This reagent was removed, wells washed, and tetramethylbenzidine added as substrate. Optical density was measured using a microplate reader (680XR, Bio-Rad, Hercules, and CA) at wavelength 450 nm. The PEDF concentrations (ng/ml) in each sample were estimated using internal PEDF standards.

### Western Blot analysis

The molecular weight of coPEDF was confirmed by western blot analysis using the conditions of Laemli. 100 ng proteins was resolved by SDS-PAGE and electro transferred to nitrocellulose membrane (Bio-Rad) for 2 h at 0.3A. Membranes were washed with 0.1% Tween-TBS (TBS-T) buffer (3×, 10 min each) then treated with TBS-T buffer containing 5% dry milk for 2 h at room temperature and subsequently incubated with an anti PEDF polyclonal antibody (1∶1000; made in our lab) overnight at 4°C. Blots were then incubated with goat anti rabbit conjugated IgG (1∶1000) (Bio-Rad) for 1 h at room temperature and antibody binding visualized using the Imun-Star HRP Substrate Kit (Bio-Rad) followed by exposure to BIOMAX MR FILM (Kodak).

### Mass Spectrometry

coPEDF and wtPEDF were analyzed by mass spectrometry (HMC Molecular Genetics Core Facility at PSU) to confirm protein identity. Approximately 100 nM (10 µl) of the protein was reduced with 20 µl of 2 mM TCEP (Tris(2-carboxyethyl)phosphine, Sigma #C4706) in 25 mM ammonium bicarbonate (NH_4_HCO_3_) (pH 8.0) at 37°C for 15 min. This was followed by alkylation with 20 µl of 20 mM iodoacetamide in 25 mM NH_4_HCO_3_. The solution was then incubated in the dark at 37°C for 30 min and dried. Samples were trypsinized with 27 µl of 0.02 µg/µl of Promega Sequencing grade modified trypsin (Promega, Madison, WI) in 10% acetonitrile, 40 mM NH_4_HCO_3_, pH 8.0 overnight at 48°C and then evaporated to dryness. Bicarbonate was removed from the sample by resuspension in 200 µl water and evaporated to dryness. This was repeated three times followed by a final resuspension in 250 µl water and evaporation to ∼10 µl volume. 1 µl of 1% trifluoroacetic acid solution in water was added, peptides desalted, concentrated using ZipTip SCX tips (Millipore, Billerica, MA), and eluted directly onto stainless still target plates (Applied Biosystems, Framingham, MA). The sample spots were dried and overlaid with 0.7 µl of the MALDI matrix, a-cyano-4-hydroxycinnamic acid, (Sigma) (5 mg/ml in 50% acetonitrile, 2 mg/ml NH_4_HPO_4_) and then air-dried at room temperature. The peptide mixtures were analyzed by mass spectrometry using an Applied Biosystems (ABI) 4800 Proteomics MALDI TOF/TOF Analyzer (Foster City, CA). Protein identification was carried out using the Mascot algorithm version 1.9 (Matrix Science, London, UK) as implemented in the GPS Explorer analysis program (version 3.5, Applied Biosystems) against nonredundant protein database and Swissport (version 2005.0315). Peptide mass fingerprints and MS/MS spectra with a confidence interval of >95% were used as protein identification criteria [37].

### UV and circular dichroism (CD) spectroscopy

CD measurements of coPEDF and wtPEDF were performed with a JASCO J-75 spectropolarimeter (Jasco, Easton, MD) and the far UV CD spectra were obtained in the range of 210–260 nm at 10°C. The temperature was controlled using a water bath thermostat RTE-111 (Neslab, Instruments, Portsmount, NH). Each spectrum was the result of the average of three individual scans after subtraction of the buffer spectra. The protein concentrations used for each sample was 0.2 −0.4 mg/ml in 20 mM NaH_2_PO_4_, 1 mm DTT, pH 7.5 buffer. A path length of 1 mm barrel quartz cell was used in all experiments. The proteins molar ellipticity was calculated as [⊝] = (⊝_0_*Mw/A)/10*L*C, where ⊝_0_, is the experimental ellipticity; Mw, the molecular mass of protein; A, the number of amino acid residues, C, the protein concentration in the solution in milligrams per milliliter; and L, the optical path length in centimeters. UV spectrum was taken using a U-2001 Spectrophotometer (Hitachi) and a 1cm rectangular quartz cuvette.

#### Neurotrophic assays Neurite outgrowth

Human neuroblastoma SHSY5Y cells (ATCC, Mannassas, VA) were used to examine the neurite outgrowth effects of coPEDF as has been reported for wtPEDF [1]. The cells were routinely maintained in DMEM supplemented with 2% fetal bovine serum (FBS; Atlanta Biologicals, Norcross, GA) and 1% penicillin-streptomycinneomycin (PSN; Invitrogen, Carlsbad, CA). For the neurite outgrowth experiments, SHSY5Y were seeded onto glass coverslips in 24-well culture plates at a density of 1×10^4^ cells per well. The cells were adapted to SF-MEM for 1 h, then treated with wtPEDF (100 ng/ml) or coPEDF (100 ng/ml) in SF-MEM for 5 d. Control cells were maintained in SF-MEM alone. Neurite outgrowths were visualized in live cultures by light microscopy. Samples were also fixed using 4% paraformaldehyde for 1 h at room temperature and incubated with PBS containing 5% BSA (PBS/BSA). The samples were then immunolabeled with polyclonal rabbit anti-neurofilament 200 kDa (1∶1000; Sigma, St Louis, MO) overnight at 4°C, followed by incubation with goat anti-rabbit cy3 (1∶1000; Jackson ImmunoResearch, West Grove, PA), FITC-conjugated phalloidin (1∶1000; Invitrogen) and Hoescht 33258 (1∶10,000; Sigma) for 1 h at room temperature and immunolabel visualized by confocal microscopy (Fluoview FV1000 confocal microscope, Olympus, Center Valley, PA). Six microscopic fields for each treatment were imaged and the neurite length of 20 cells in each field measured using the Image J software (NIH). The histogram shows the average neurite length of 3 independent experiments (mean ± standard deviation (S.D.).

#### Oxidative stress assay

The protective effects of coPEDF and wtPEDF against hydrogen peroxide (H_2_O_2_) toxicity were determined in cultured neuroblastoma SHSY5Y exposed to oxidative stress using the lactate dehydrogenase (LDH) cytotoxicity assay (Roche, Indianapolis, IN, USA). Cells were seeded in 96-well culture plates at a density of 1×10^4^ cells per well, adapted to serum-free MEM (SF-MEM) for 1 h, then treated with PEDF (100 ng/ml) or coPEDF (100 ng/ml) in SF-MEM for 24 hr. Control cultures were maintained in SF-MEM alone. The cultures were then challenged with H_2_O_2_ (160 or 320 µM) for 24 h. In some experiments, the cultures were first treated with 600 µM H_2_O_2_ for 1 hr after which time the H_2_0_2_ was removed and the cultures treated with wtPEDF or coPEDF for 24 hr. The percentage of cell death was estimated by measuring the LDH activity in the RPE conditioned media.

#### Endothelial Tube formation assay

24-well plates were pre-coated with Matrigel (BD Biosciences), and HUVEC cells (1×105 cells/well) were seeded onto coated plates in the presence of VEGF (3 ng/ml). The cultures were supplemented with one of the following: wtPEDF (100 ng/ml), coPEDF (100 ng/ml), or the non-antiangiogenic peptide PEDF_78–121_ (25 ng/ml) to determine effects of these molecules on endothelial tube formation. After incubation for 8 h at 37°C, tube formation was assessed in the cultures and images taken using a phase contrast microscope (10×) with an attached CCD camera.

### Results Cloning of artificially synthesized PEDF with bacteria preferred codons

**Figure 1:**
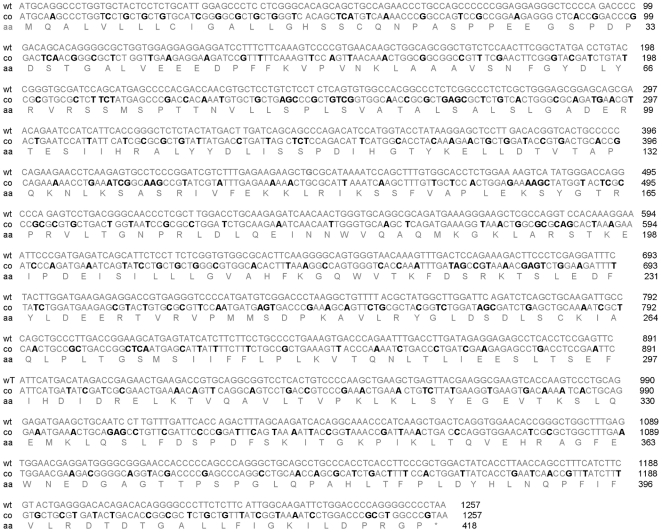
Sequence comparison between wt and coPEDF genes. wt: wild-type; co: codon-optimized; aa: amino acid.

**Figure 2:**
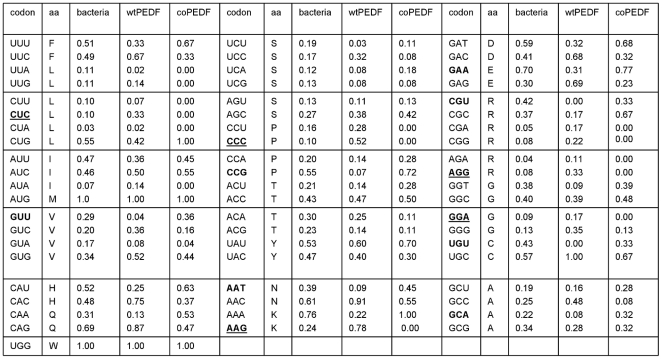
Comparison of codon usage in E coli, wtPEDF, coPEDF. The figure shows the codon changes made in synthetic PEDF (examples are in bold type and underlined) and the frequency balance adjustments of codon usage between the host and coPEDF (examples are in bold type).

The efficiency of heterologous PEDF production in bacteria can be greatly diminished by codon bias usage. Here we optimized expression of the human wtPEDF gene using bacteria preferred codons according to the GeneOptimizer software algorithm (GeneScript, CA) for expression in the pET32a vector and presented the comparative sequence in Figure 1. In the engineered human PEDF gene there were replacements of several rare codons to those favored by bacteria for example, CUC to CUG (L), CCC to CCG (P), AAG to AAA (K), AGG to CGC (R). There is also a better balance in the frequency of usage for several codons between the host and coPEDF, for example GUU (V), CAU (H), CCG (P), AAT (N), GAA (E), CGU (R), UGU (C), GCA (A) (Figure 2). The increase in coPEDF expression most likely reflects these changes.

**Figure 3:**
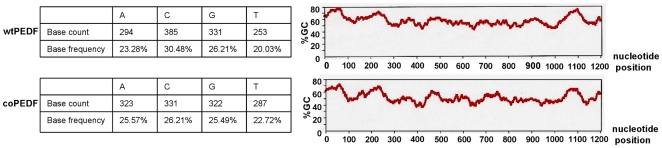
Base composition and GC% content of wtPEDF and coPEDF.

**Figure 4:**
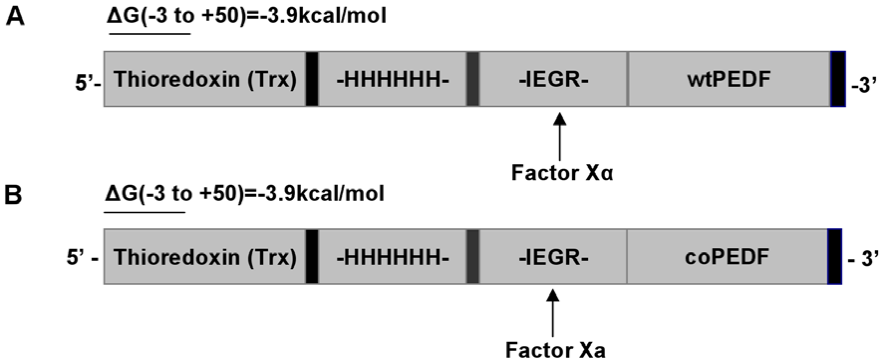
pET32a constructs for wtPEDF and coPEDF protein expression in *E.coli*. The figure shows wtPEDF and coPEDF inserts, upstream thioredoxin (Trx) fragment, factor Xα cleavage site, and mRNA folding energy of 3.9 kcal/mol at the Trx start site (−3 to +50) of the fusion proteins.

**Figure 5:**
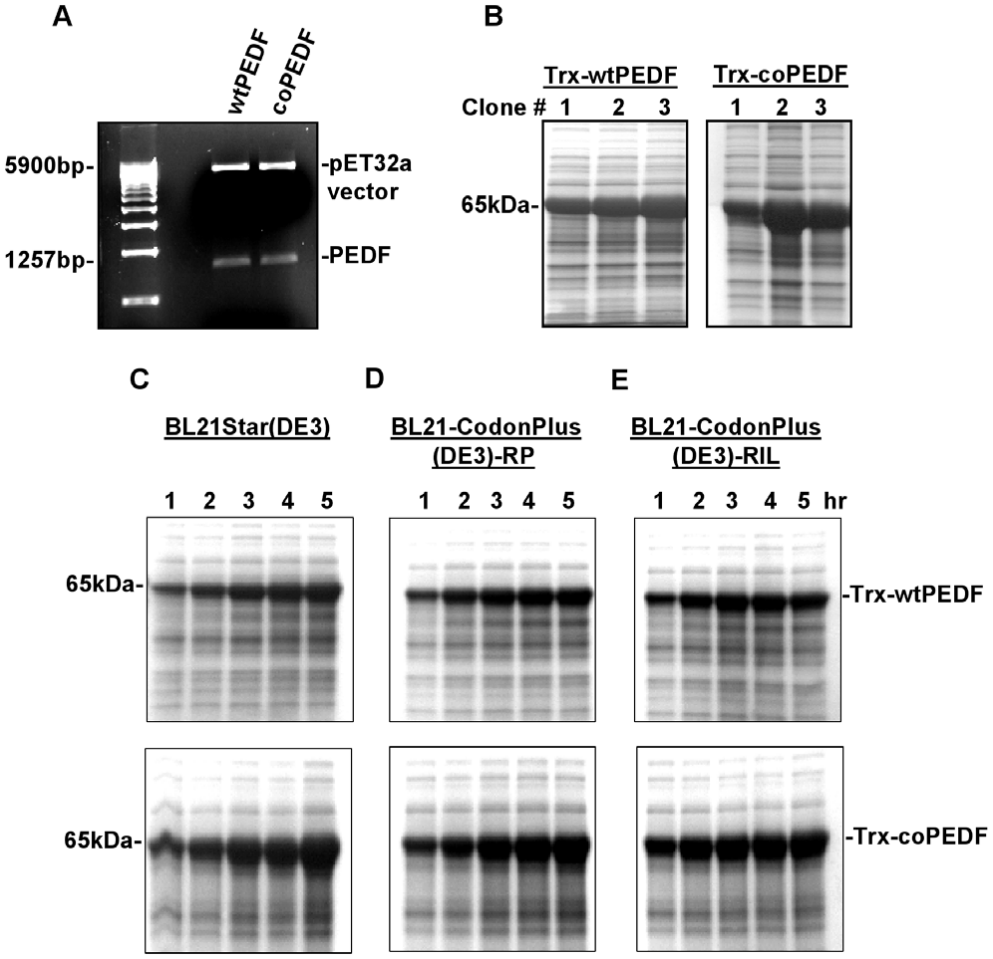
Expression of Trx-PEDF in bacteria. A. PCR gel showing the expected product insert size of 1257 bp for both wtPEDF and coPEDF and the pET32a vector size. B. Comassie blue stained (CB) SDS gels showing lysates from BL21Star(DE3) colonies expressing the Trx-wtPEDF or Trx-coPEDF fusion proteins at 65 kDa. Clone #3 and #2 were selected for high expression yields of the proteins. C.D.E. SDS gels showing expression levels of Trx-wtPEDF and Trx-coPEDF fusion proteins with IPTG induction over 1–5 hr in BL21Star(DE3), and two codon-bias-adjusted host cells: BL21-CodonPlus(DE3)-PR and BL21-CodonPlus(DE3)-RIL cells.

The base composition and GC% content of native and synthetic PEDF cDNA sequences differ by 9.97% and 4.99%, respectively (Figure 3) but there were no changes in the amino acid sequence between the two proteins. Codon optimized PEDF was successfully assembled using overlapping PCR strategy and the wtPEDF and coPEDF genes cloned into the pET32a vector with an appended thioredoxin gene fragment of 109 amino acid sequence to improve protein folding, 6 His-tag residues for ease of purification of the expressed protein, and a factor Xa Trx.tag sequence for easy cleavage of the 50 kDa PEDF protein (Figure 4). PCR gel confirmed the expected product insert size of 1257 bp for both wtPEDF and coPEDF and a high molecular weight band of 5900 bp representing the pET32a vector (Figure 5A). After transformation of *E. coli* BL21Star (DE3) with the constructs, several positive bacterial colonies were identified and two clones (clone #3 for Trx-wtPEDF and clone #2 for TrxcoPEDF) were selected for further analysis because of high expression yields (Figure 5B). The proteins are shown to migrate at a molecular weight of 65 kDa since they include the upstream 15 kDa thioredoxin sequence (Figure 5B).

### Quantitative yields of Trx-wtPEDF and Trx-coPEDF

**Figure 6:**
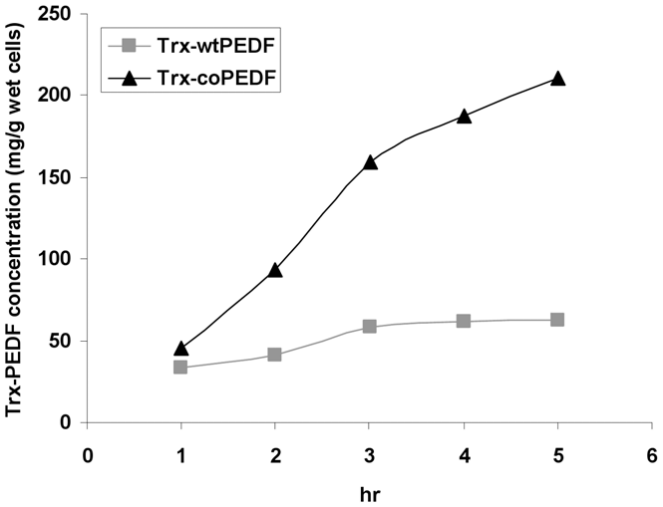
Time course expression yields for Trx-wtPEDF and Trx-coPEDF fusion proteins in BL21Star(DE3) lysate.

Expression of the recombinant proteins in BL21Star(DE3) was maximally induced with 0.5 mmol/L IPTG at 25°C for 5 hrs as shown in the time course study in Figure 5C. The two constructs were also expressed in the BL21-CodonPlus(DE3)-RIL cells expressing the rare tRNA*^argU,ileY,leuW^* genes, which recognize the AGA/AGG, AUA, and CUA codons, respectively and the BL21-CodonPlus(DE3)-RP cells expressing extra copies of the tRNA*^argU,proL^* genes, which recognizes the AGA/AGG and CCC codons, respectively (Figure 5D,E). At all time points, there was improved protein expression of the synthetic PEDF gene in native and codon-bias adjusted host cells compared to the native gene in bacterial lysates (Figure 5C,D,E). Using ELISA and densitometry methods, the yields for Trx-wtPEDF and TrxcoPEDF in all host lysates after 5 h expression were calculated and presented in Table 1. While there was an insignificant increase in both Trx-wtPEDF (∼9% and 15%) and and Trx-coPEDF (1% and 5%) in hosts supplied with extra copies of rare tRNA genes, the ratio of expression was 3 to 3.4 fold greater yield of the codon-optimized gene to the wt gene product in both conventional and codon-bias-adjusted hosts (Table 1). Quantitative yields for the proteins in BL21Star(DE3) host cell lysates between 1–5 hr expression are presented in Figure 6 and table 2. At all comparable time points, the yields of Trx-coPEDF were greater than those for the wt gene. The highest expression levels were observed after 5 hr IPTG induction, where the amount of Trx-coPEDF was 485 mg/L (212.7 mg PEDF/g wet *E coli*) and Trx-wtPEDF, 142 mg/L (62.1 mg PEDF/g wet *E. coli*), representing an approximate 4× greater yield of the gene product with codon optimization (Table 2). No significant growth differences in the bacterial cultures were observed at OD 600 nm after transfection of the plasmids.

**Table 1:** Quantitative yields of fusion proteins in bacteria lysates after 5 h expression using densitometry and ELISA assays.

	mg/L		mg/g wet cells	
	Trx-wtPEDF	Trx-coPEDF	Trx-wtPEDF	Trx-coPEDF
BL21Star(DE3)	142	485	79.33	242.5
BL21-Codonplus(DE3)-RP	154	507	76.24	266.84
BL21-Codonplus(DE3)-RIL	163	488	80.30	256.84

A small increase in expression of both proteins was observed in hosts supplemented with extra copies of rare tRNA genes but in all cases, coPEDF expression was approximately 3–3.5 fold greater than wtPEDF.

**Table 2:** Comparative yields of Trx-wtPEDF and Trx-coPEDF in BL21Star(DE3) lysates.

	mg/L		mg/g wet cells	
hr	Trx-wtPEDF	Trx-coPEDF	Trx-wtPEDF	Trx-coPEDF
1	77	103	33.6	45.2
2	95	212	41.5	93.0
3	132	363	57.9	159.2
4	141	428	61.7	187.7
5	142	485	62.1	212.7

Codon preference modifications of the PEDF gene resulted in greater translational efficiency of the fusion proteins.

### Characterization of expressed coPEDF

**Figure 7:**
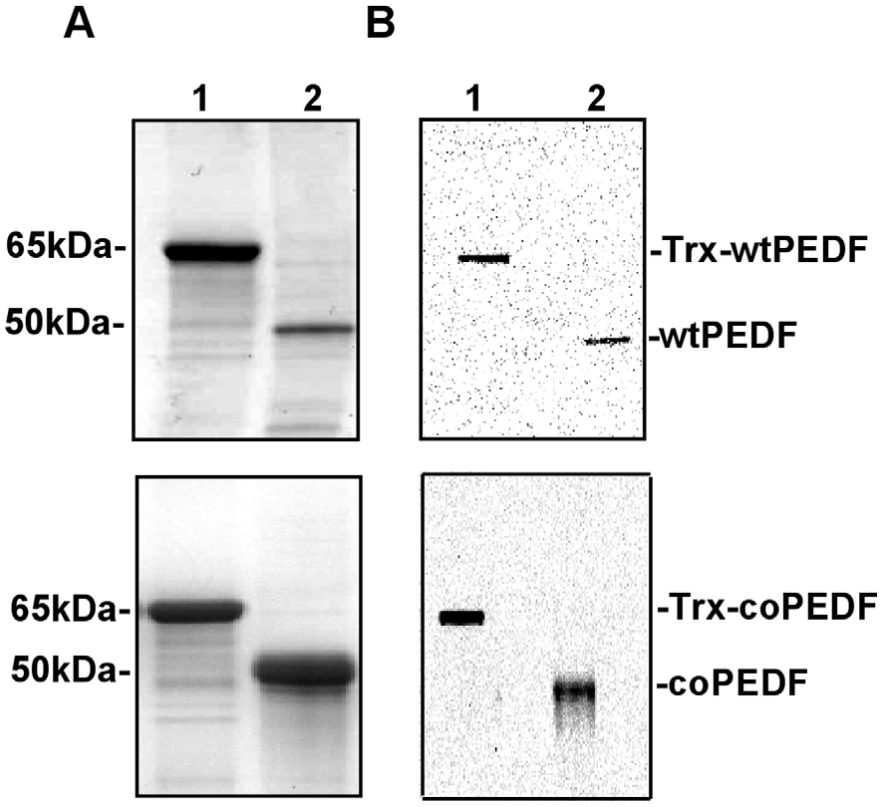
Purification of PEDF. A. Comassie blue (CB) stained SDS gels showing: lane 1: purified Trx-PEDF fusion protein (65 kDa); lane 2: cleaved PEDF (50 kDa). B. Western Blots (WB) using a PEDF polyclonal antibody confirms PEDF protein identity in both the fusion and cleaved products. Lane 1: Purified Trx-PEDF fusion proteins (65 kDa); lane 2: cleaved PEDF (50 kDa).

Purification of the proteins by Ni-NTA metal-affinity chromatography followed by cleavage from the N-terminal thioredoxin with factor Xα resulted in the expected 50 kDa size for wtPEDF and coPEDF as shown in the comassie blue stained gel in Figure 7A (Lane 1: uncleaved protein; lane 2: cleaved protein). The molecular sizes of the proteins were confirmed by western blot analyses using a PEDF polyclonal antibody generated in our lab as shown in Figure 7B (Lanes1, 2: uncleaved and cleaved proteins respectively).

We obtained 94.1 mg/L (41.27 mg/g wet cells) purified coPEDF (50 kDa) and 26.5 mg/L (11.62 mg/g wet cells) wtPEDF (50 kDa) by expressing the pET32a constructs in BL21Star(DE3) (Table 3). In comparison, the purified yields of native PEDF expressed in pEV-BH [38], PET41a [39], pET22b [40], and yeast Pichia Pastoris [41] were 2.6, 3.8, 10, and 20 mg/L (Tables 3, 4), respectively, representing a 36 -5 fold increase in coPEDF expression yields. There with also a ∼10, 7, 3 fold increase of wtPEDF using the pET32a vector compared to wtPEDF yields using pEV-BH, pET41a, and pET22b, respectively and a 1.3 fold increase over the yeast expression system (Tables 3, 4).

**Table 3:** Purified protein.

	mg/L	mg/g wet cells
wtPEDF(pET32a)	26.5	11.62
coPEDF(pET32a)	94.1	41.27

Quantitative measurement indicates a ∼4 fold greater yield of purified cleaved coPEDF protein over the native PEDF.

**Table 4:** Comparative purified yields of wtPEDF from published reports.

wtPEDF(pEV-BH)	wtPEDF(pET41a)	wtPEDF(pET22b)	Yeast
2.6 (1.3 mg/g wet cells) [38]	3.8 [39]	10 [40]	20 [41]

The comparisons indicate an ∼5–36 fold greater yield of the coPEDF protein in all host expression systems tested over wtPEDF [38]. Becerra et.al., JBC 1993, 268:23148 [39]. Zhang et.al., Biotechnol Lett 2005, 27:403 [40]. Wang et.al., Prep Biochem Biotechnol. 2006, 36:127 [41]. Sanchez et.al., J of Biotechnol 2008, 134:193.

**Figure 8:**
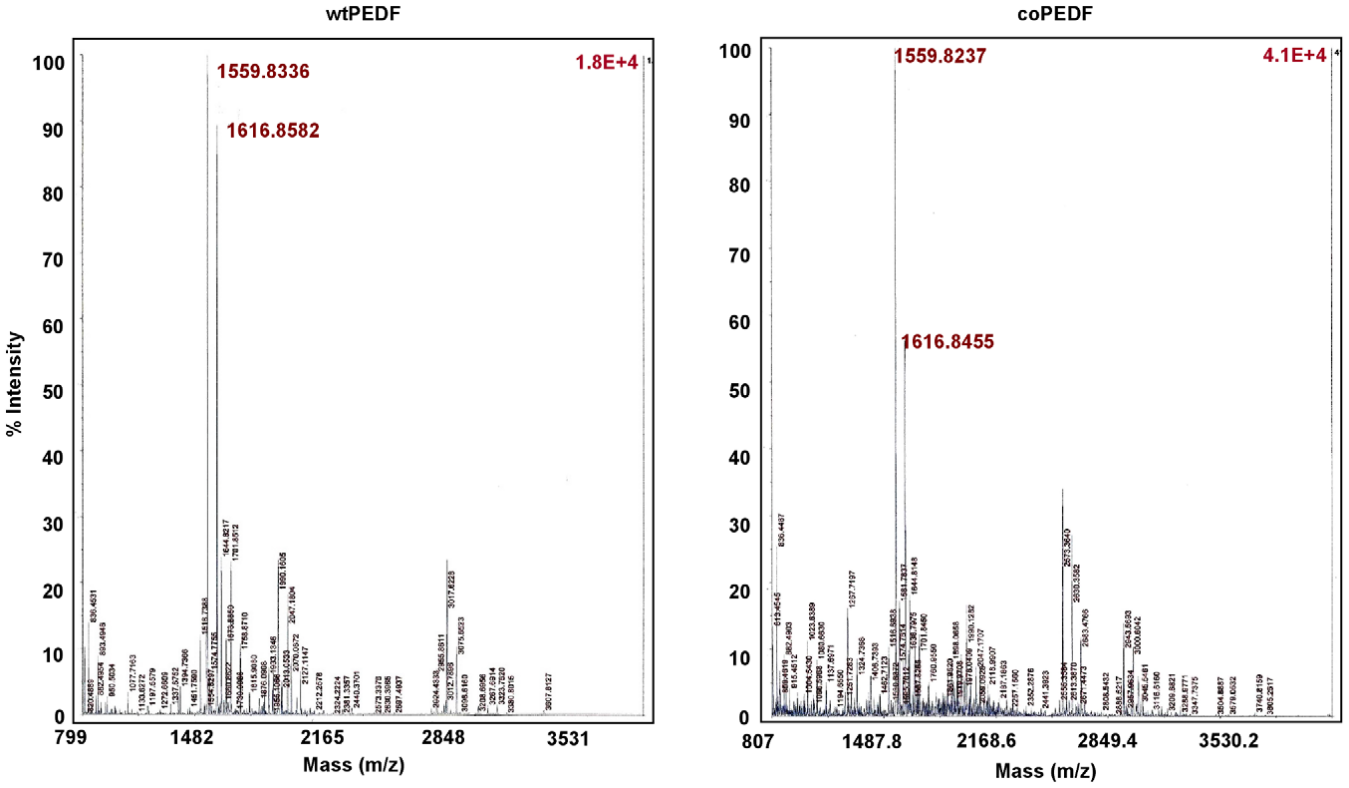
MALDI-TOF analysis confirms that the wtPEDF and coPEDF proteins are identical.

**Figure 9:**
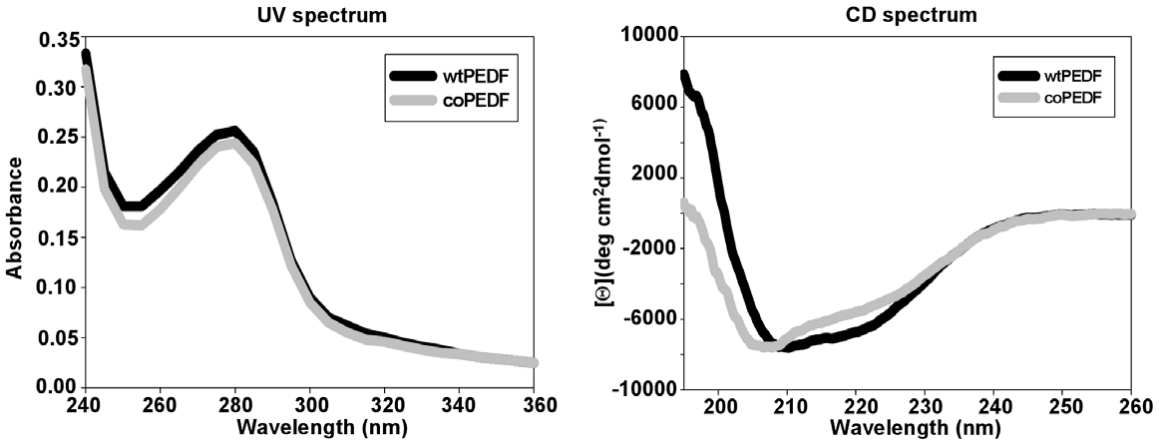
Structural identity comparisons by UV and CD spectral analyses. The profiles show that coPEDF and wtPEDF have similar UV and CD spectra.

The coPEDF and wtPEDF proteins identity was confirmed by gene sequencing and Maldi-TOF analyses (Figure 8) and by circular dichroism (CD) spectroscopy (Figure 9), which monitors global changes in the protein secondary structure [42]. Far UV and CD spectra recordings indicated that bacteria preferred codon usage did not alter the secondary structure of coPEDF and that the molecules are α/β proteins.

### RNA folding energies

Using the “mfold” RNA folding program, we calculated the free energy of 5′ translated Trx-coPEDF and Trx-wtPEDF mRNA at (−3 to +50) of the Trx start site. The calculations indicate that the 5′ regions of both native and engineered fusion genes were identical and contained mRNA structures with a ΔG of −3.9 kcal/mol. This excludes the mRNA folding energy hypothesis for increased translational yields of Trx-coPEDF.

### coPEDF retains full biological activity as wtPEDF

**Figure 10:**
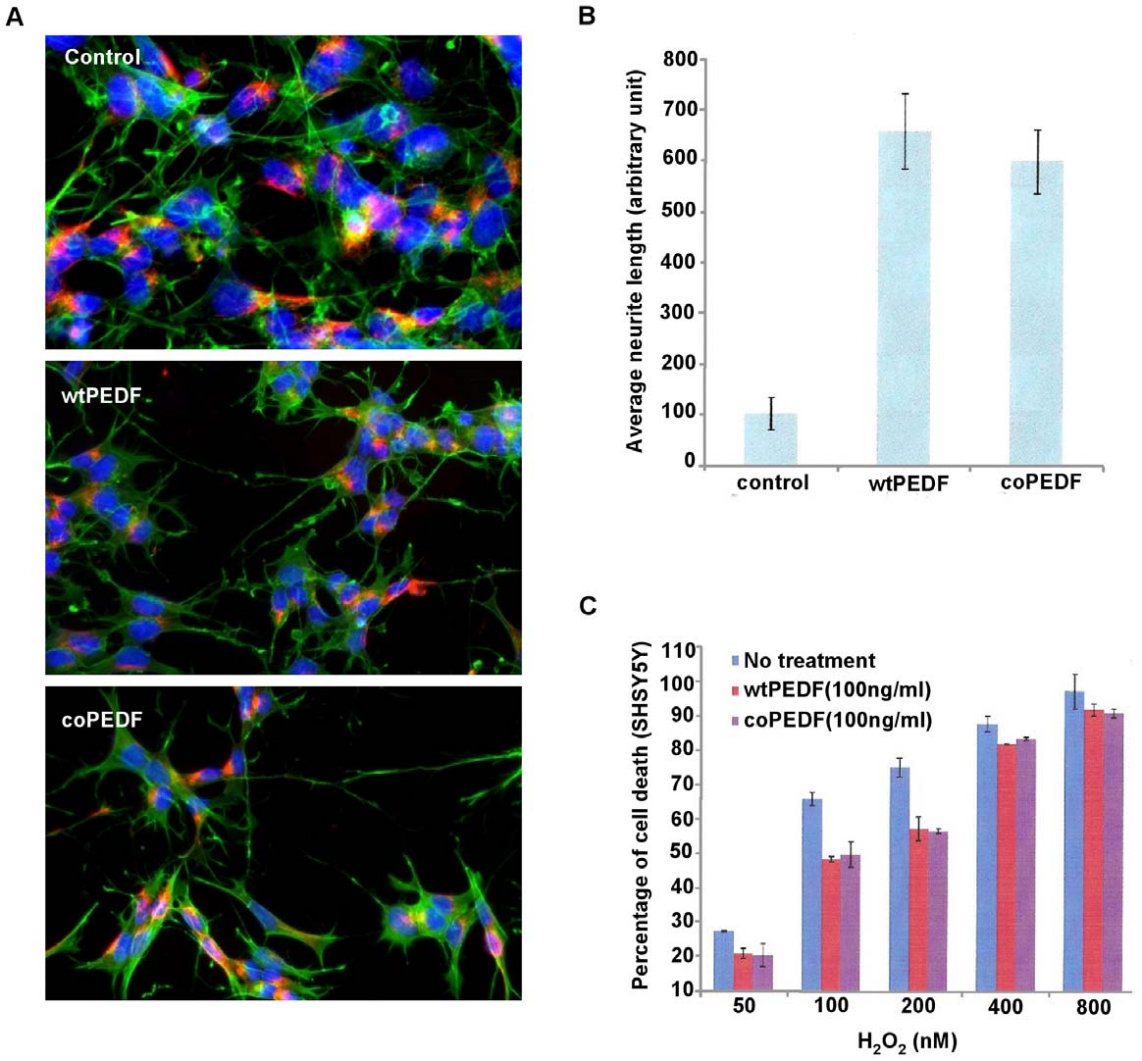
coPEDF promotes neurite outgrowth in SHSY5Y neuroblastoma cells and protects cells from hydrogen peroxide toxicity. A. Cells treated with wtPEDF or coPEDF for 5 d were fixed and immunolabeled with antibodies against neurofilaments (red) and β-actin (green) proteins, and stained with DAPI, (blue). B. The histogram represents the mean neurite length (+/− SD). C. Neuroprotective effects were observed for wtPEDF and coPEDF proteins when cells were treated with H_2_O_2_ for 24 hr. The LDH assay histogram shows that maximal effects were achieved with 100 ng/ml of either wt or coPEDF proteins when cells were exposed to 200 nM H_2_O_2_ (n = 3).

**Figure 11:**
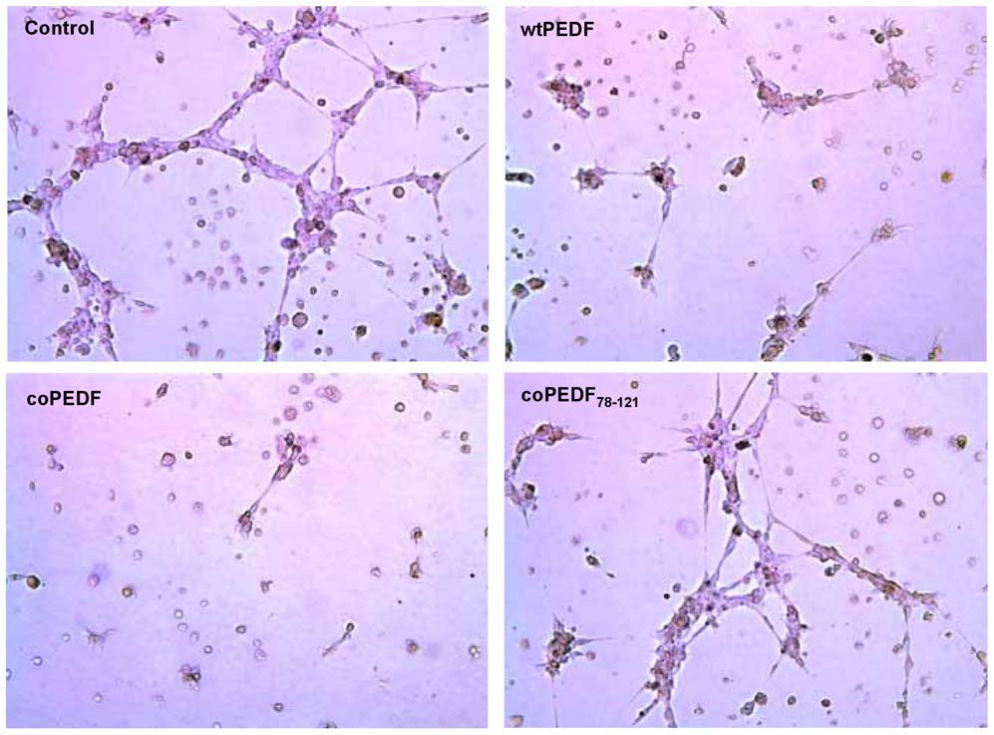
coPEDF blocks endothelial tube formation. The anti-angiogenic activity of coPEDF are illustrated by endothelial tube-formation assays on human umbilical vein endothelial cells (HUVEC) grown in Matrigel-coated plates in the presence of wtPEDF, coPEDF, a neuroprotective PEDF_78–121_ peptide control or no treatment control. The endothelial sprouting and tubes formed were observed after 6 hr of treatment. (n = 3).

Native PEDF is a potent neurotrophic and neuroprotective agent [1]. Here we show that both wt and coPEDF proteins induced neurite outgrowth from SHSY5Y neuroblastoma cells (Figure 10A). The neurite lengths were 5.9±0.63 and 6.6±0.74-fold longer when cells were cultured with either coPEDF or wtPEDF, respectively, compared to non-treated controls (Figure 10B). The neurites exhibited well-branched structures and expressed the neurofilament 200 kDa protein as detected by immunolabelling. This finding supports previous data that PEDF promotes neurite outgrowths in neuroblastoma cells, another neural derived tumor. wtPEDF and coPEDF were equally effective in protecting the neuroblastoma cells from hydrogen peroxide toxicity with a maximal 30% reduction in cell death when cells were exposed to 200 µM H2O2 (Figure 10C). We also showed that coPEDF was equally as effective in blocking endothelial tube formation (63% inhibition compared to control; n = 3) as the native protein (Figure 11), which is not surprising since the amino acid sequences of wt and coPEDF proteins are identical (Figure 1). In this experiment we also showed that the neurotrophic PEDF peptide (PEDF_78–121_), which served as a negative control in this study, had negligible effects on endothelial tube formation. These data provide evidence that codon bias improves yield of biologically active PEDF and is a useful strategy for large-scale production of this molecule for further biochemical and functional analyses of the protein and for translational research.

## Discussion

An alternative method for obtaining high yields of PEDF has been an unmet need in the field of PEDF neuroregenerative medicine. The objective of this work was to improve expression of PEDF in *E coli* to obtain cost effective production of the protein for translational research.

Heterologous production of recombinant mammalian proteins can be quite challenging since these genes often contain expression limiting regulatory elements and codons infrequently used outside their original context [31], [34], [43]. The degeneracy of the genetic code allows many amino acids to be encoded for by more than one synonymous codon and the same protein by many alternative nucleic acid sequences. The frequency with which a given codon is used in the genetic code can vary significantly among organisms but how an organism develops preferences for specific codons is a vigorously disputed topic in molecular evolution. There is consensus though, that codon preference correlate with cognate isoacceptor tRNA concentrations available in the natural host environment and reflects a balance necessary for efficiency of translation. [31], [34], [43]–[45].

While *E. coli* is still the most widely used host for expressing mammalian genes, codon usage space maps indicate that it is not an optimal host for expressing proteins encoded for by human codon usage because of the significant divergent codon bias that exists between the two organisms [34]. In heterologous protein expression, ribosomes pause when encountering a rare codon and can detach from the mRNA and abort translation. Forced expression of rare codon-containing genes is likely to exhaust endogenous pools of the analogous tRNAs, and lead to growth arrest, premature termination of transcription and/or translation, decrease mRNA stability, and increased frameshifts, deletions, and misincorporations. Heterologous expression then, may not only result in reduced protein yields but lead to a mixture of expressed proteins with altered function and increased immunogenicity, a major consideration in translational research [34], [46]–[49].

Although resolving codon bias is challenging, many have shown that heterologous protein expression can be improved by engineering foreign genes to preferentially use codons compatible with the host without altering the protein or by supplying extra copies of rare tRNA genes to host cells [50]–[54]. Both approaches have shown success in dramatically improving heterologous protein expression [34], [49], [55]–[59]. In some cases though, rare codon substitutions with more favorable host codons can result in protein misfolding, loss of biological function, and have a significant impact on protein expression [49], [60]–[63].

Here, we used both strategies and showed that expression of the human PEDF protein is greatly improved by engineering the native gene to be compatible with *E coli* codon bias usage. Supplying rare tRNA to *E. coli* hosts had a small but insignificant effect on expression of both native and synthetic PEDF genes. Protein yields were more robust with the redesigned gene in both conventional and codon bias-adjusted hosts compared to native PEDF suggesting that optimizing the gene for bacterial codon usage improved speed and efficiency of translation in this host.

Some have posited that translational efficiency is influenced by the stability of the mRNA folding near the ribosomal binding site of the protein and that codon bias has a net positive effect on the overall fitness of host cells [64] while others argue that both are important to translational efficiency [65]. In this case, however, the native and synthetic PEDF mRNA folding energies were identical and unlikely to contribute significantly to the differences in translation efficiency observed. Both genes were expressed as fusion proteins with the 109 amino acid upstream thioredoxin gene and their ΔGs were −3.9 kcal/mol at the Trx translational start site.

Native PEDF yields were greater by ∼3 to 10 times more over previously reported yields in bacterial hosts and expression levels were similar in yeast [38]–[41]. The translational advantage of wtPEDF in our expression system is most likely due to differences in the vector used and an appended Trx sequence, which is known to improve protein folding and translational efficiency [66]. We did not observe a significant difference in expression of human Trx-PEDF between codon-bias-adjusted hosts and conventional *E. coli* hosts when the cells were transformed with the pET32a wtPEDF or coPEDF constructs.

Regardless of the comparably higher yields of native PEDF to other published expression systems, the synthetic codon optimized gene resulted in protein levels that were ∼5 to 36 fold greater than amounts reported for the native gene [38]–[41] and a ∼3 fold greater yield in both codon-bias-adjusted and conventional E. coli host cells transformed with the pET32a constructs. One might argue that increasing host intracellular tRNA diversity increases the metabolic load of actively growing organisms in a way that has a negative impact on expression of a few proteins and/or that specific bacterial proteins may repress the expression of some rare tRNA genes [67]. The increase in coPEDF over wt PEDF expression in conventional host is most likely due to the replacement of specific codons in the gene with those favored by the host and by balancing the frequency with which some codons are used between the two systems. Addition of rare tRNA genes to host cells did not boost wtPEDF levels, suggesting that, in this case, a wider range of rare tRNA genes may be necessary to achieve the levels obtained for the synthetic gene. While expanding the tRNA diversity may be a good strategy to increase translational efficiency, fidelity and function of the protein may be compromised. As expected, there was no significant augmentation in coPEDF expression in the codon-bias-adjusted host cells. One possible explanation for this is that the extra tRNA genes expressed in these cells recognized codons that were replaced in the synthetic gene. Our findings suggest that while protein yields can be influenced by choice of expression vectors, 5′ mRNA folding energies, or by increasing tRNA diversity in host cells, redesigning some mammalian genes to use preferred host codons may increase translational efficiency.

Not only was the codon bias adjusted gene expressed at higher levels, the function of the protein remained intact, an important consideration in clinical applications. Using both biophysical and biochemical methods, we confirmed that the synthetic and wild type gene products expressed in native *E. coli* competent cells were identical and by functional assays validated the neurotrophic and antiangiogenic efficacies of the proteins. An important finding from this study is that the differentiation actions of PEDF on tumor cells is not restricted to retinoblastoma [1] but may be a general biological function of the protein on actively proliferating neural tumors. It is tempting to speculate that the neurite promoting activity of the protein on neural tumor cells is a consequence of its actions in reducing oxidative stress. We also showed that the antiangiogenic potency of the engineered gene product is intact and comparable to the native protein in endothelial tube formation assays. These findings have implications for the use of coPEDF in attenuating neural tumor growth and metastasis by blocking angiogenesis and promoting tumor cell differentiation and in neuroregenerative medicine where axonal growth and neurite survival/regeneration are essential to restore function.

In summary, heterologous PEDF expression is greatly improved by using codons that match those preferred by the host translational system without altering protein function. Large-scale production of this protein is certainly an attractive approach to facilitate the speed at which therapeutic efficacy can be validated in larger animal models of tumor growth, angiogenesis, and neurodegenerations without the drawbacks of viral mediated transfer of the gene.
